# Practical Notes and Formulæ

**Published:** 1881-09-20

**Authors:** 


### PRACTIACL NOTES AND FORMULÆ.

**Listerine.**—Dr. H. A. Cottell, Demonstrator of Anatomy in the Louisville University, in a published letter, says: I had an experience with Listerine the other day which gave me no little comfort. I had ordered it as a throat wash in a case of scarlet fever where it did good work; but the nurse, mistaking it for another medicine, gave a tablespoonful of it to the patient, who was an adult. The mistake was soon discovered, and the father of the patient made haste to a neighboring drug store where, through telephone, he informed me of the situation, and no one but a physician, who has had experience with poisoning through misadventure, can appreciate the satisfaction with which I assured him that the wash was non-poisonous, and could do no harm. Had I used carbolic acid, or almost any other antiseptic, I might have had a case of poisoning on my hands, or at least, great trouble in antidoting the toxic drug. It is needless to say that no unpleasant symptoms followed the mistaken dose, which was given at about half-past five in the afternoon. It may have contributed to a favorable result, for the patient slept well during the following night, awaking in the morning refreshed and improved.

Though, theoretically, Listerine ought to be of service in a zymotic affection like scarlet fever, it will not do to lay much stress upon the apparent good effect of an isolated dose, accidentally given, but too much cannot be said in favor of an efficient antiseptic that proves itself to be innocuous in any dose likely to be taken through mistake.

Prof. E. R. Palmer, who was associated with me in this case, is pleased with the qualities of Listerine, and confirms the statements and conclusions above stated. I believe that this antiseptic will prove itself worthy of high favor with the profession.

**Metastatic Prostatitis.**—Dr. R. T. McConnell, of Alabama, writes: Having noticed an article in the July issue of your Journal, on Epidemic Metastatic Parotiditis, soliciting more information on the subject, I propose giving a history of 42 cases occurring in my practice. Of this number 66⅔ per cent. have had metastasis; also 23⅔ per cent. had remittent fever coming on just previous to, or during inception of mumps. These cases were males from 12 to 78 years of age. Metastatic parotiditis has been more severe in this immediate community than elsewhere in this country. Whether or not the remittent condition attending mumps in this section influences metastasis, I cannot satisfactorily determine; however, from my record, I am of that opinion. Heretofore, metastasis was said to have been produced by over-exertion, exposure, etc., but the majority of my cases have not been caused by any such influences. I hope this article will elicit further information on the subject.

**Antiseptic Treatment of Enteric Fever.**—In the British Medical Journal, Dr. J. E. Shelly reports early and rapid fall of temperature, retardation and steadying of the pulse, improvement in stools, cleaning of tongue, early removal of abdominal tenderness, refreshing sleep, and remarkable general comfort, without complications, in a series of cases of typhoid fever treated by the formula of Rothe, somewhat modified. He gave every two, three or four hours a draught containing one or two minims of carbolic acid, one to three minims of tincture of iodine, half a drahm of simple syrup, and an ounce of lemon-water till apyrexia was produced, and after that at longer intervals for several weeks. Like Dr. Rothe, he has had good results from the same combination in choleraic and autumnal diarrhea and diphtheria.—*Louisville Med. News.*

**Rhus Aromatica in Gonorrhea.**—Dr. J. C. Spiegel reports, in Therapeutic Gazette, a case of gonorrhea successfully treated with the following:

| | | | |
|---|---|---|---|
| ℞ Ext. rhus aromat. fluid............ | aa fl. ℥ss; | 16.00 | Gm., |
| Glycerinæ...................... | | | |
| Aquæ............................ | ℥j; | 32.00 | " |

M. Sig. For injection three times daily after urinating. Relief was immediate and cure complete.—*Louisville Med. News.*

**The Treatment of Anorexia.**—M. Huchard has had frequent opportunities of administering the following prescription to patients in whom it is necessary to stimulate the appetite:

| | |
|---|---|
| Water................................ | Gm. 250 (℥viij), |
| Peppermint water.................. | aa Gm. 10 (ʒijss), |
| Tincture of gentian................ | |
| Tincture of bitter orange peel........ | |
| Tincture of stellate anise seed........ | |
| Compound tincture of cardamons........... | Gm. 3 (♏ xlv), |
| Bitter drops of balsam.................... | Gm. 2 (ʒ ss). |

Filter. Teaspoonful to be taken after each meal.—*Le Progres Med. Practitioner.*

**Night Sweats.**—

| | | | |
|---|---|---|---|
| ℞ Acid sulphurici..................... | ʒijss; | 10.00 | Gm., |
| Tinct. opii.......................... | ʒj; | 4.00 | " |
| Syrupi aurantii.................... | ℥j; | 30.00 | " |
| Aquæ, ad....................... | ℥viij; | 240 00 | " |

Sig. Two tablespoonfuls three times a day.—*Farquharson.*

The above prescription is also very useful in summer diarrhœa, and as a prophylactic against painter's colic.—*Med, Gaz.*

**Ulceration of the Os.**—In hyperplastic swelling of the vaginal portion, and in follicular ulcers of the os, Dr. Kisch, of Berlin, has derived great use from it (Berliner Klinische Wochenschrift, No. 42, 1879). He employs—

| | |
|---|---|
| ℞ Iodoformi............................ | ʒj, |
| Glycerinæ............................ | ʒx, |
| Olei menthæ piper...................... | gtt. vi–x. M. |

Shake well together. Steep a plug of cotton wool in this and apply to the vaginal portion.—*Med. and Surg. Rep.*

